# Identification and Classification for the *Lactobacillus casei* Group

**DOI:** 10.3389/fmicb.2018.01974

**Published:** 2018-08-22

**Authors:** Chien-Hsun Huang, Shiao-Wen Li, Lina Huang, Koichi Watanabe

**Affiliations:** ^1^Bioresource Collection and Research Center, Food Industry Research and Development Institute, Hsinchu, Taiwan; ^2^Molecular Medicine Research Center, Chang Gung University, Taoyuan, Taiwan; ^3^Department of Animal Science and Technology, College of Bioresources and Agriculture, National Taiwan University, Taipei, Taiwan

**Keywords:** *Lactobacillus casei* group, MALDI-TOF MS, MLSA, species-specific PCR, whole genome sequence

## Abstract

*Lactobacillus casei, Lactobacillus paracasei*, and *Lactobacillus rhamnosus* are phenotypically and genotypically closely related, and together comprise the *L. casei* group. Although the strains of this group are commercially valuable as probiotics, the taxonomic status and nomenclature of the *L. casei* group have long been contentious because of the difficulties in identifying these three species by using the most frequently used genotypic methodology of 16S rRNA gene sequencing. Long used as the gold standard for species classification, DNA–DNA hybridization is laborious, requires expert skills, and is difficult to use routinely in laboratories. Currently, genome-based comparisons, including average nucleotide identity (ANI) and digital DNA–DNA hybridization (dDDH), are commonly applied to bacterial taxonomy as alternatives to the gold standard method for the demarcating phylogenetic relationships. To establish quick and accurate methods for identifying strains in the *L. casei* group at the species and subspecies levels, we developed species- and subspecies-specific identification methods based on housekeeping gene sequences and whole-cell matrix-assisted laser desorption/ionization time-of-flight mass spectrometry (MALDI-TOF MS) spectral pattern analysis. By phylogenetic analysis based on concatenated housekeeping gene sequences (*dnaJ, dnaK, mutL, pheS*, and *yycH*), 53 strains were separated into four clusters corresponding to the four species: *L. casei, L. paracasei* and *L. rhamnosus*, and *Lactobacillus chiayiensis* sp. nov. A multiplex minisequencing assay using single nucleotide polymorphism (SNP)-specific primers based on the *dnaK* gene sequences and species-specific primers based on the *mutL* gene sequences provided high resolution that enabled the strains at the species level to be identified as *L. casei, L. paracasei*, and *L. rhamnosus*. By MALDI-TOF MS analysis coupled with an internal database and ClinProTools software, species- and subspecies-level *L. casei* group strains were identified based on reliable scores and species- and subspecies-specific MS peaks. The *L. paracasei* strains were distinguished clearly at the subspecies level based on subspecies-specific MS peaks. This article describes the rapid and accurate methods used for identification and classification of strains in the *L. casei* group based on housekeeping gene sequences and MALDI-TOF MS analysis as well as the novel speciation of this group including *L*. *chiayiensis* sp. nov. and ‘*Lactobacillus zeae*’ by genome-based methods.

## Introduction

*Lactobacillus* is the largest genus of the family *Lactobacillaceae*. As of May 2018, it consists of 196 validly published species^1^, and these species are commonly isolated not only from environments associated with fermented food, such as fruits, meat, sourdough, vegetables, and wine, but also from the gastrointestinal and vaginal tracts of humans and animals (Watanabe et al., 2008; Hammes and Hertel, 2009; Pot et al., 2014; Tamang et al., 2015). Currently, lactobacilli are widely applied in fields related to food, feed, pharmaceuticals and biotechnology; for example, they are used as dairy starters, probiotics, vaccine carriers and silage inoculants (Seegers, 2002; Giraffa et al., 2010), which are among the most economically interesting applications of lactic acid bacteria (LAB). Lactobacilli are gram-positive, rod-shaped, facultatively anaerobic or microaerophilic, non-spore-forming, acid-tolerant, and catalase-negative bacteria with DNA G+C content that is usually less than 50 mol%. *Lactobacillus casei, Lactobacillus paracasei*, and *Lactobacillus rhamnosus* are phylogenetically and phenotypically closely related; together, they are regarded as the *L. casei* group. Members of this group are facultatively heterofermentative, have 45–47 mol% DNA G+C content, and have identical peptidoglycan types (L-Lys-D-Asp) (Salvetti et al., 2012). The widely known probiotic strains that are part of this group (such as *L. casei* strain Shirota and *L. rhamnosus* GG) are used worldwide in fermented dairy products or food supplements and as probiotics to enhance host health (Shida and Nomoto, 2013; Ashraf and Shah, 2014; Reid, 2015; Orlando et al., 2016). Although this group comprises many commercially valuable strains, its taxonomic status has long been contentiousas has its nomenclaturebecause methods with inadequate taxonomic resolution have been used, leading to species being mislabeled in products, publications and some publicly available DNA sequences.

The taxonomy of the *L. casei* group has been altered numerous times. In the Approved Lists of Bacterial Names (Skerman et al., 1980), *L. casei* described as a single species with five subspecies on the basis of phenotypic features: *L. casei* subsp. *casei, L. casei* subsp. *alactosus, L. casei* subsp. *pseudoplantarum, L. casei* subsp. *tolerans*, and *L. casei* subsp. *rhamnosus*. On the basis of DNA–DNA homology, this species was reclassified into three species and two subspecies (Collins et al., 1989): (i) *L. casei* (including strains previously belonging to *L. casei* subsp. *casei*); (ii) *L. paracasei* comprising two subspecies, *L. paracasei* subsp. *paracasei* (including the previous subspecies *L. casei* subsp. *alactosus* and *L. casei* subsp. *pseudoplantarum*) and *L. paracasei* subsp. *tolerans* (including the previous subspecies *L. casei* subsp. *tolerans*); and (iii) *L*. *rhamnosus* (including the previously classified subspecies *L. casei* subsp. *rhamnosus*).

The conventional taxonomy of the genus *Lactobacillus* has mostly depended on morphological, physiological, and biochemical traits, and scientists have principally depended on commercial identification kits, such as, the API 50 CHL system (Charteris et al., 2001; Boyd et al., 2005). Although phenotypic tests have been applied to determine the metabolic characteristics of each strain, the *L. casei* group members have numerous characteristics in common that result in remarkably similar phenotypes. This similarity in phenotypic traits may be influenced by the possible acquisition or depletion of plasmids encoding large number of carbohydrate fermentation traits or environmental conditions, and this may result in isolates with metabolic features that are atypical (Ahrné et al., 1989), rendering the established identification methods laborious and imprecise. Therefore, using molecular taxonomic methods to improve species identification of *Lactobacillus* strains has become common among researchers.

On the basis of analyses of cellular soluble protein patterns, randomly amplified polymorphic DNA (RAPD) fingerprinting, and DNA–DNA hybridization (DDH), Dellaglio et al. (1991) and Dicks et al. (1996) proposed that *L. casei* subsp. *casei* ATCC 393 and ‘*Lactobacterium zeae*’ ATCC 15820 should be reclassified as ‘*Lactobacillus zeae*’ nom. rev., designated the strain ATCC 334 as a neotype of *L. casei* subsp. *casei*, and forbade the name *L. paracasei*. In 2008, the Judicial Commission of the International Committee on Systematics of Prokaryotes (ICSP) stated that the current taxonomy of the *L. casei* group is comprised of three closely related species: *L*. *casei* (type strain: ATCC 393), *L. paracasei* subsp. *paracasei* (type strain: ATCC 25302) and *L. paracasei* subsp. *tolerans* (type strain: ATCC 25599), and *L. rhamnosus* (type strain: ATCC 7469) (Tindall, 2008). Huang et al. (2018) updated the taxonomy proposing a new species for the *L. casei* group based on whole-genome sequencing (WGS), multilocus sequence analysis (MLSA), matrix-assisted laser desorption/ionization time-of-flight mass spectrometry (MALDI-TOF MS), phenotypic characterization and species-specific PCR.

As far as the molecular taxonomic methods, such as RAPD-PCR (Huang and Lee, 2009) and temporal temperature gradient gel electrophoresis (TGGE) (Vásquez et al., 2001) have been applied to identify *L. casei* group strains. DDH has long been used as a method for delineation of bacterial species because of its status as a conventional gold standard genotypic assay (Wayne et al., 1987; Stackebrandt et al., 2002). However, DDH is laborious, requires expert skills, and is inconsistently reproducible. Furthermore, the data obtained are non-cumulative and difficult to apply to all microorganisms (Mehlen et al., 2004; Pontes et al., 2007). Recent progress in technology for DNA sequencing technology has enabled genome sequences to be applied relatively inexpensively, providing a high-quality and adequate bioinformatic tool for taxonomic studies of prokaryotes (Rosselló-Móra and Amann, 2015). Analyzing comparative sequencing of the 16S rRNA gene is currently a common route to identification and classification of bacteria. Since 1994, strains with similarity of more than 97% to the 16S rRNA gene sequence have been considered to belong to the same species (Stackebrandt and Goebel, 1994). The species-level cutoff percentage was evaluated to 98.7% and then 98.65% in 2006 and 2014, respectively (Stackebrandt and Ebers, 2006; Kim et al., 2014). However, in many cases, species that are closely related—such as those belonging to the *Lactobacillus buchneri* group (*L. buchneri, L. kefiri, L. parabuchneri*, and *L. parakefiri*), *L. casei* group (*L. casei, L. paracasei*, and *L. rhamnosus*), *Lactobacillus plantarum* group (*L. fabifermentans, L. plantarum, L. paraplantarum*, and *L. pentosus*) and *Lactobacillus sakei* group (*L*. *curvatus, L. graminis*, and *L. sakei*) are indistinguishable using 16S rRNA gene sequencing because of the high degree of similarity (as high as 99%) of the 16S rRNA gene sequences among the species (Torriani et al., 2001; Koort et al., 2004; Watanabe et al., 2009; Huang et al., 2010; Huang and Lee, 2011). Consequently, substantially conserved protein-encoding genes are useful as alternative molecular targets for distinguishing closely related species. Thus, a housekeeping sequence in addition to supplemental techniques including PCR-restriction fragment length polymorphism (RFLP), SNaPshot minisequencing, species- and subspecies-specific PCR, MLSA, and MALDI-TOF MS, has successfully been applied to achieve higher resolution within the *L. casei* group (Felis et al., 2001; Naser et al., 2007; Watanabe et al., 2008; Huang and Lee, 2009, 2011; Huang et al., 2011, 2014, 2015, 2018; Bottari et al., 2017).

Huys et al. (2006) reported that more than 28% of commercially available probiotic products were labeled incorrectly at the genus or species level because methods that limit taxonomic resolution were used. Correct identification of probiotic strains at the species level is essential for safety assessment as it allows a linkage to potentially relevant, species-related scientific, and technological information. Therefore, a method that quickly and accurately identifies starter cultures is required for the management and quality control of commercial probiotic products.

This article describes the rapid and accurate methods for identification and classification of strains in the *L. casei* group based on housekeeping gene sequences and MALDI-TOF MS analysis in addition to the current state in classification of the *L. casei* group, including a novel species identified using genome-based methods.

### *L. casei* Group Housekeeping-Gene-Based Phylogenetic Analysis

Housekeeping genes exhibit high sequence variation and are feasible alternatives to the 16S rRNA gene in accurately classifying and identifying bacteria (Petti, 2007). For example, gene sequence analysis of the phenylalanyl t-RNA synthase alpha subunit (*pheS*) and RNA polymerase alpha subunit (*rpoA*) has been used to distinguish closely related LAB species of the genera *Lactobacillus, Enterococcus, Leuconostoc, Pediococcus*, and *Weissella* (Naser et al., 2005, 2007; De Bruyne et al., 2007, 2008, 2010; Chao et al., 2010, 2012, 2013; Oki et al., 2012; Nguyen et al., 2013; Nyanzi et al., 2013; Huang et al., 2018). To date, numerous protein-encoding genes for example, *dnaJ, dnaK, hsp60, mutL, pheS, recA, rpoA, spxB, tuf*, and *yycH* have been used as phylogenetic targets to discriminate among species of the *L*. *casei* group and have exhibited satisfactory resolution with a high level of discrimination (Felis et al., 2001; Naser et al., 2007; Huang and Lee, 2011; Huang et al., 2014, 2015; Yu et al., 2014; Sardaro et al., 2016; Bottari et al., 2017). However, horizontal gene transfer (HGT) or lateral gene transfer may cause problems in phylogenetic tree construction based on a single gene. Concatenation of several housekeeping genes may reduce the weight of HGT and recombination (Gogarten et al., 2002; Boucher et al., 2004; Macheras et al., 2011; Timilsina et al., 2015). Furthermore, it could accurately locate taxonomic positions for closely related species and strains (Glaeser and Kämpfer, 2015).

The *ad hoc* committee aimed at re-evaluation of the species definitions in bacteriology has determined that sequencing of several housekeeping genes (i.e., five or more) is a reliable method of examining deep-level phylogenies and complex species groups (Stackebrandt et al., 2002). MLSA based on housekeeping gene sequences provides high discriminative power and has the potential to replace DDH (Zeigler, 2003; Gevers et al., 2005; Vandamme and Peeters, 2014; Glaeser and Kämpfer, 2015). However, MLSA has several drawbacks; for example, no universal threshold for species definition, no common set of genes has yet been suggested, and few reference sequences have been released through public databases (Mulet et al., 2010; Lai et al., 2014; Li C. et al., 2014; Rosselló-Móra and Amann, 2015; Peeters et al., 2016; Liu et al., 2017). Therefore, MLSA must be improved to render it more feasible and applicable.

Multilocus sequence analysis has been increasingly used to identify and describe novel species (Killer et al., 2014, 2017; Chen et al., 2017; Doi et al., 2017; Liu et al., 2017; Ribeiro et al., 2017; Modesto et al., 2018). Mattarelli et al. (2014) stated that a minimum of two additional phylogenetic markers should be used to accurately identify isolates, especially for describing novel species in the genera *Bifidobacterium* and *Lactobacillus*.

**FIGURE 1:**
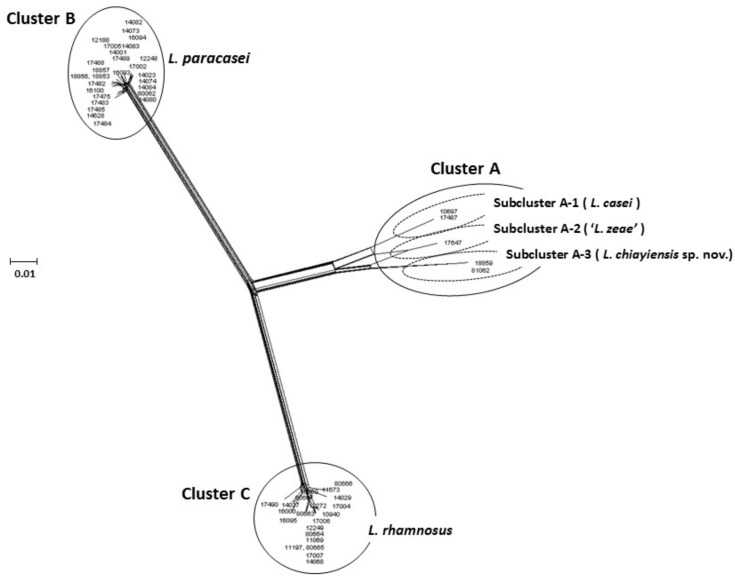
Concatenated split network tree based on five genes. The *dnaJ, dnaK, mutL, pheS*, and *yycH* gene sequences (2567 bp) from 53 *L. casei* group strains were concatenated and reconstructed using the SplitTree4 program. Sequence similarities were corrected using the Jukes-Cantor correction. Bar, 0.01 expected nucleotide substitutions per site.

Huang et al. (2018) used MLSA to characterize the *L. casei* group based on concatenated sequences of three housekeeping genes (*dnaK, pheS*, and *yycH*; 1627 bp), with the neighbor-joining, maximum-likelihood, and minimum-evolution methods used for phylogenetic analysis. The results showed that a novel species, *L. chiayiensis* sp. nov. (strains BCRC 81062^T^ and BCRC 18859), comprised an independent cluster notably distinct from *L. casei* and ‘*L. zeae*.’ Furthermore, a split network tree based on the concatenated sequences of five housekeeping genes (*dnaJ, dnaK, mutL, pheS*, and *yycH*; 2567 bp) was performed, and the 53 strains of the *L. casei* group were divided into three principal clusters: Cluster A (comprising five strains of *L. casei*, ‘*L. zeae*,’ and *L. chiayiensis* sp. nov.); Cluster B (comprising 27 strains of *L. paracasei*); Cluster C (comprising 21 strains of *L. rhamnosus*). Split network analysis revealed that Cluster A comprised three subclusters: subclusters A-1 (*L. casei*), A-2 (‘*L. zeae*’), and A-3 (*L. chiayiensis* sp. nov.) (**Figure 1**). Thus, multilocus phylogenetic analysis of the sequence data of ‘*L. zeae*’ indicated that this microorganism could be reclassified as an independent species or a subspecies of *L. casei*.

### Development of Species and Subspecies-Specific Primers

#### Detection Through Conventional PCR Assay

The bacterial 16S rRNA gene has nine hypervariable regions (V1–V9). These nine regions exhibit substantial sequence diversity among bacteria. The hypervariable regions contain species-specific sequences that are suitable for diagnostic assays (Van de Peer et al., 1996). Ward and Timmins (1999) have developed three PCR primer pairs that are specific for the *L. casei* group member (*L. casei, L. paracasei*, and *L. rhamnosus*) on the basis of variation in the V1 region of the 16S rRNA gene. These primer pairs were validated using 63 *Lactobacillus* isolates from cheeses. However, ambiguous results were obtained when these primers were used to analyze 76 wild isolates from dairy samples (milk and differently ripened cheeses) (Bottari et al., 2017). A species-specific primer for ‘*L. zeae*’ was designed by Desai et al. (2006) on the basis of the 16S rRNA gene. As well as the 16S rRNA gene, the 16S–23S rRNA intergenic spacer region was investigated to successfully identify *L. paracasei* and *L. rhamnosus* (Song et al., 2000). Protein-coding genes evolve rapidly to provide greater interspecies variation and can act as supplemental targets for designing species-specific primers. We previously developed several highly specific primer sets targeted the *dnaK, mutL*, and *yycH* genes for the species-level identification of *L. casei, L. chiayiensis* sp. nov., *L. paracasei*, and *L. rhamnosus* by using PCR-based methods (Huang et al., 2014, 2018; Huang and Huang, 2018). Studies have established a multiplex species-specific PCR assay on the basis of the 16S–23S rRNA spacer region and *tuf, mutL*, and *pepR* genes for identifying *L. casei* group members (Ventura et al., 2003; Kwon et al., 2004; Watanabe et al., 2008; Bottari et al., 2017).

Primer specificity for the selected species can be advanced only if known gene sequences exist that possess particular and satisfactory variations in nucleotides. By contrast, novel species-specific primers can be developed on the basis of sequence characterized amplified region (SCAR) markers through RAPD-PCR analysis. SCARs are detected by PCR using specific primers designed based on the sequence data of RAPD fragment. Multiplex PCR assay of SCAR makers is useful method for reducing the costs and time for routine identification. A combination of RAPD-PCR and the SCAR technique has been applied to develop the species- and subspecies-specific primers for *L. paracasei* subsp. *tolerans, L. rhamnosus*, and ‘*L. zeae*’ within the *L. casei* group (Huang and Lee, 2009).

#### Detection Using SNaPshot Minisequencing Assay

SNaPshot is a primer extension-based multiplex system for detecting single nucleotide polymorphisms (SNPs) by using fluorescently labeled dideoxynucleotides (ddNTPs). Each fluorescent ddNTP emits a unique wavelength, which is subsequently represented as a particular color in a base-dependent manner. The extension products with fluorescent labeling can be visualized using capillary electrophoresis with automated sequencing. Huang et al. (2011) designed a PCR primer pair specific to *L. casei* group by using *rpoA* gene sequence and performed a SNaPshot minisequencing assay to identify species belonging to the *L. casei* group. A SNaPshot minisequencing assay using *dnaK* as a target gene was also developed, and five species-specific SNP primers were subsequently designed by analyzing the conserved regions of the *dnaK* gene sequences. The specificity of this assay was evaluated using 63 *L. casei* group strains. SNaPshot minisequencing assay combined with group-specific PCR was confirmed to quickly, accurately, and inexpensively identify the *L. casei* group at the species level. By using species-specific PCR based on *dnaJ* gene sequences with SNaPshot minisequencing, strains of *L. rhamnosus* were identified with high accuracy at the species level; furthermore, the method determined the SNP haplotypes (Huang et al., 2015).

Multiplex minisequencing is a method for determining the exact nucleotide located at a particular site, particularly when numerous SNPs are screened simultaneously within a single reaction tube. Only 40 min is required for minisequencing products to undergo automated fluorescent capillary electrophoresis, making this method quicker than direct sequencing. A total of 2.5 h is required for sequencing products to undergo capillary electrophoresis.

### Rapid Identification and Classification of *L. casei* Group Strains at the Species and Subspecies Levels Using MALDI-TOF MS

MALDI-TOF MS is a phenotype-based method that can be used for the identification, classification, and dereplication of large numbers of microorganisms on the basis of the specific proteomic profiles of these microorganisms (Ghyselinck et al., 2011). Applied in routine clinical diagnosis, and its use has been extended to environmental monitoring, food safety, biodiversity, and gut microbiology (Pavlovic et al., 2013; Samb-Ba et al., 2014; Santos et al., 2016). MALDI-TOF MS can typically discriminate between bacterial strains at both the genus and species levels using proteomics-based identification, and subspecies- and strain-level taxonomic resolution is possible when the method is combined with specific mass spectral (MS) peaks (Spinali et al., 2015). MALDI-TOF MS is straightforward, rapid, accurate, and inexpensive (although the initial outlay for the instrument is considerable), and it has high throughput (Neville et al., 2011; Sandrin et al., 2013; Tran et al., 2015). However, reference databases are mainly designed for routine clinical practice (Bizzini and Greub, 2010), and expansion of the database is necessary to increase identification rates through matching. In addition, and as a disadvantage associated with this method, procuring fresh, pure cultures and sufficient bacteria cells (10^5^–10^7^ cells) are required for protein extraction and analysis.

MALDI-TOF MS has been effectively used for identifying strains of lactobacilli isolated from dairy and meat products (Angelakis et al., 2011; Doan et al., 2012; Dušková et al., 2012; Nacef et al., 2017), fermented food (Kim et al., 2017), carious dentin from children (Callaway et al., 2013), human oral cavities and vaginas (Anderson et al., 2014), and poultry (Dec et al., 2014). A reliance on protein fingerprint profiling only could produce unclear findings for some closely related species or subspecies (e.g., *Bacillus pumilus* and *Bacillus safensis, Lactobacillus johnsonii* and *Lactobacillus gasseri*, and *Lactobacillus plantarum* subspecies *plantarum*, and *Lactobacillus plantarum* subspecies *argentoratensis*) (Šedo et al., 2013; Branquinho et al., 2014; Wieme et al., 2014). Thus, researchers recognize that particular MS peaks must be identified and developed for diagnosis. MS peaks from bacterial cell lysate consist mainly of ribosomal proteins and include several housekeeping proteins, such as the housekeeping genes used for MLSA to determine high-level taxonomy and phylogeny (Welker and Moore, 2011). A ribosomal protein typing-based method using MALDI-TOF MS has been proposed, and since then, it has been used to successfully differentiate *Bacillus* spp., *Bifidobacterium animalis, Bifidobacterium longum, Lactococcus lactis*, and the *L. casei* group (Tanigawa et al., 2010; Hotta et al., 2011; Sato et al., 2011, 2012; Ruiz-Moyano et al., 2012).

Huang and Huang (2018) used a MALDI-TOF MS instrument (Microflex LT, Bruker Daltonics) to rapidly identify *L. casei* group strains at the species and subspecies-level by using ethanol/formic acid/acetonitrile for protein extraction in a routine protocol (Chambers et al., 2015) with MS peaks analyzed using ClinProTools software (Bruker Daltonics). Type strains of the *L. casei* group were used to construct main spectral profiles (the highest quality spectra) for compiling an in-house database given the optimized culture conditions [cultivated anaerobically at 37°C on de Man, Rogosa and Sharpe (MRS) agar for 20 h]. The reference strains of *L. casei* group were used to validate the in-house database. Forty-eight (100%) strains had high scores (mean: 2.45 ± 0.1) and were accurately identified at the species level, which was consistent with the results obtained using genotypic methods (housekeeping gene sequencing and species-specific PCR).

During a study whose objective was to isolate lactobacilli from different environmental samples, strain BCRC 81062^T^ was isolated from cow manure collected in Chiayi, Taiwan. Strain BCRC 18859 (=NRIC 1947) was isolated from coconut juice from the Philippines, which was distributed by the NODAI.

Research Institute Culture Collection (NRIC; Japan) as *L. paracasei* subsp. *tolerans*. These two strains could not be clearly identified as any recognized species of the genus *Lactobacillus* by 16S rRNA gene sequencing analysis.

**FIGURE 2:**
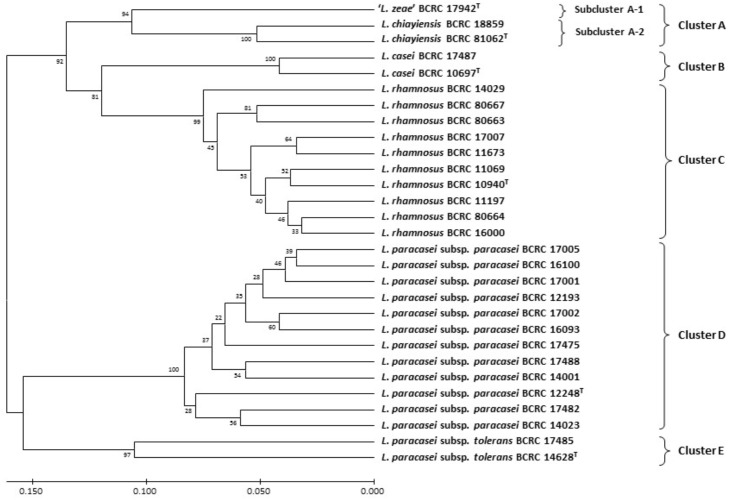
Dendrogram derived from UPGMA cluster analysis of MALDI-TOF MS spectra in the 2000–9500 *m/z* region (224 peaks) of strains in the *L. casei* group. Bootstrap values (%) based on 1000 replicates are given at the nodes. Bar, proportion of different peaks.

Huang et al. (2018) performed the MALDI-TOF MS analysis for classification of these two novel strains. A dendrogram derived from UPGMA cluster analysis of MALDI-TOF MS spectra in the 2000–9500 m/z (224 peaks) region of strains in the *L. casei* group were divided into five distinct clusters: Cluster A comprised two novel *L. chiayiensis* strains (BCRC 81062^T^ and BCRC 18859) and the ‘*L. zeae*’ strain; Cluster B comprised two strains of *L. casei*; Cluster C comprised 10 *L. rhamnosus* strains; Cluster D comprised 12 *L. paracasei* subsp. *paracasei* strains; Cluster E comprised two *L. paracasei* subsp. *tolerans* strains. Cluster A was divided into two subclusters: ‘*L. zeae*’ (BCRC 17942^T^) was the single strain located in independent subcluster A-1. Two strains of *L. casei* were located in subcluster A-2 (**Figure 2**). This result was in good concordance, with the exception of the subspeciation of *L. paracasei* in the MLSA analysis based on housekeeping gene sequences. In addition, species- and subspecies-specific MS peaks were observed in the MS profiles, and these peaks served as the specific markers for differentiating *L. casei* group strains at both species and subspecies levels (**Table 1**).

**Table 1:** Species- and subspecies-specific MALDI-TOF MS peaks used for differentiation of the *Lactobacillus casei* group.

Peak mass (*m/z*)	*L. chiayiensis* sp. nov. (*n* = 2)	*L. casei* (*n* = 2)	‘*L. zeae*’ (*n* = 1)	*L. rhamnosus* (*n* = 10)	*L. paracasei* subsp. *paracasei* (*n* = 12)	*L. paracasei* subsp. *tolerans* (*n* = 2)
2465	−	−	−	−	−	+
2653	−	−	−	−	+	−
3982	−	+	−	−	−	−
4112	+	−	−	−	−	−
4928	−	−	−	−	−	+
5206	−	−	+	−	−	−
5302	−	−	−	−	+	−
5571	+	−	−	−	−	−
8357	−	−	−	−	−	+
8425	−	−	−	+	−	−
10,413	−	−	+	−	−	−
11,143	+	−	−	−	−	−

*+, peak presented; −, no peak found.*

Detecting and describing taxonomic novelty and reclassifying microbes has also been accomplished using MALDI-TOF MS (Kudo et al., 2012; Wieme et al., 2012; Li L. et al., 2014; Praet et al., 2015; Yanokura et al., 2015).

### Phylogenetic Analysis of the *L. casei* Group Based on Whole-Genome Sequences

Because of the recent technological advancement in the field of WGS, dry-lab experiments using *in silico* methods based on genome-to-genome comparison can be used instead of the usual wet-lab DDH and conventional method for determining the DNA G+C content and sequencing of 16S rRNA and housekeeping genes of type strains (Richter and Rosselló-Móra, 2009; Chun and Rainey, 2014; Rosselló-Móra and Amann, 2015). WGS is now officially recognized as a source of taxonomic information (Whitman, 2016). Average nucleotide identity (ANI), digital DNA–DNA hybridization (dDDH) and tetranucleotide usage patterns (TETRA) are the most commonly used in the silico methods for species demarcation of phylogenetic relationships (Teeling et al., 2004; Goris et al., 2007; Meier-Kolthoff et al., 2013). ANI and dDDH values of 95–96% and 70%, respectively, are equivalent to DDH value of 70% and may be applied as boundaries in species delineation. In addition, dDDH values of 79–80% have been suggested for delineating subspecies within the domains Bacteria and Archaea on the basis of an investigation of genome-sequenced strains from more than 100 genera (Auch et al., 2010; Kim et al., 2014; Meier-Kolthoff et al., 2014; Richter et al., 2016). TETRA was used as an alignment-free parameter correlated with ANI because oligonucleotide frequencies are species-specific (Richter and Rosselló-Móra, 2009). Next-generation sequencing is responsible for an exponential rise in the quantity of available genome sequences of bacterial strains. However, the dubious quality of several genome sequences available in public databases (Ricker et al., 2012) is a substantial drawback. Chun et al. (2018) recommended minimal standards for utilizing genome data for taxonomic analysis of prokaryote and recommended quality requirements for genome sequences to address the aforementioned drawback.

Wuyts et al. (2017) identified three taxonomic clades (clade A: *L. paracasei*, clade B: *L. casei*, and clade C: *L. rhamnosus*) in the *L. casei* group based on a core genome phylogenetic tree, DNA G+C content analysis, and pairwise genome distances (ANIb and TETRA). These researchers also suggested that the heme-dependent catalase gene and the SOD-encoding gene play a role for molecular markers for the accurate identification of *L. casei* and *L. paracasei*.

**FIGURE 3:**
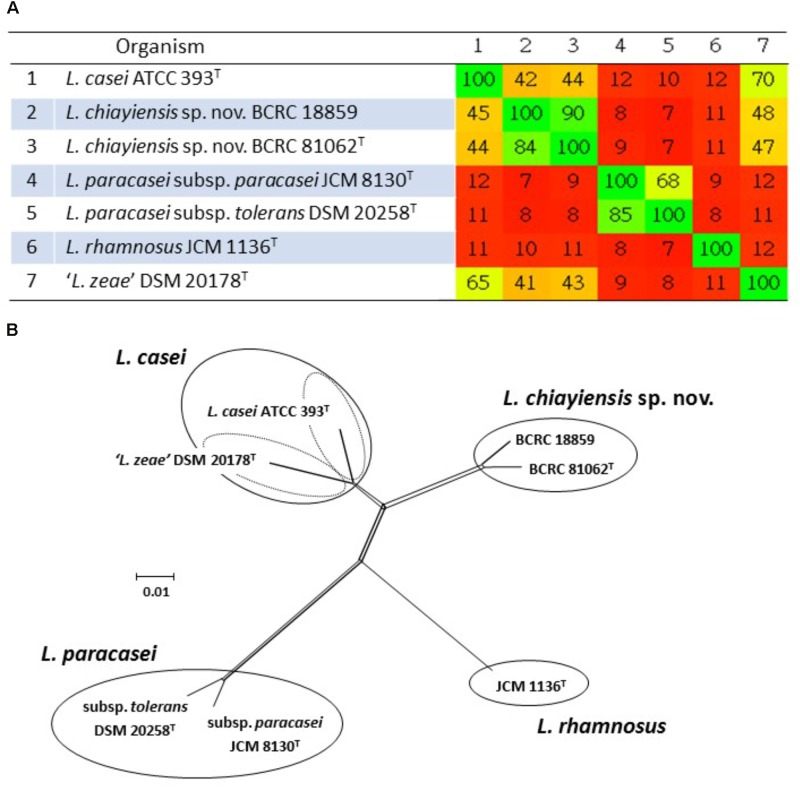
Phylogenomic analysis of the *L. casei* group. The values generated in the Gegenees software shown in the heat map indicate the percentage of similarity between the analyzed genomes; the colors varies from red (low similarity) to green (high similarity) **(A)**. The split network tree was constructed using the SplitTree4 program based on the fragmented all-against-all comparison with the Gegenees software **(B)**.

Huang et al. (2018) analyzed the draft whole genome sequences of the novel two strains (BCRC 81062^T^ and BCRC 18859). The ANI values were estimated using orthologous average nucleotide identity (OrthoANI) (Lee et al., 2016), and dDDH was performed using the genome to genome distance calculator (GGDC) with recommended Formula 2 (Meier-Kolthoff et al., 2013). The distance matrix generated using Gegenees software v.2.2.1 (Ågren et al., 2012) was plotted as a heat map, and the interspecies similarity between the novel strains and closely related type strains ranged from 7 to 44% (**Figure 3A**). A split network tree based on the fragmented all-against-all comparison of the whole genome sequences using Gegenees software to separate the novel strains and four type strains in the *L. casei* group could be separated into four clusters: Cluster A consisting of *L. casei* ATCC 393^T^ (Accession No. GCA_000829055.1) and ‘*L. zeae*’ DSM 20178^T^ (GCA_001433745.1); Cluster B consisting of two novel *L. chiayiensis* strains (BCRC 81062^T^: MSSM00000000; BCRC 18859: NOXN00000000); Cluster C consisting of *L. rhamnosus* JCM 1136^T^ (GCA_000615245.1); Cluster D consisting of *L. paracasei* subsp. *paracasei* JCM 8130^T^ (GCA_000829035.1) and *L. paracasei* subsp. *tolerans* DSM 20258^T^ (NZ_AYYJ00000000) (**Figure 3B**). The ANI and dDDH values between *L. chiayiensis* sp. nov. BCRC 81062^T^ and type strains in the *L. casei* group ranged from 77.4 to 88.5, and 22.3 to 36.1%, respectively, which were markedly lower than the commonly used cut-off threshold (i.e., 95–96% for ANI, and 70% for dDDH) for delineation of prokaryotic species. Phenotypic and chemotaxonomic characterization as well as MLSA based on three housekeeping genes (*dnaK, pheS*, and *yycH*), species-specific PCR and whole-cell MALDI-TOF MS spectral pattern analyses revealed that the novel strains (BCRC 81062^T^ and BCRC 18859) represented a single, novel species within the *L. casei* group, for which the name *L. chiayiensis* sp. nov. is proposed.

**FIGURE 4:**
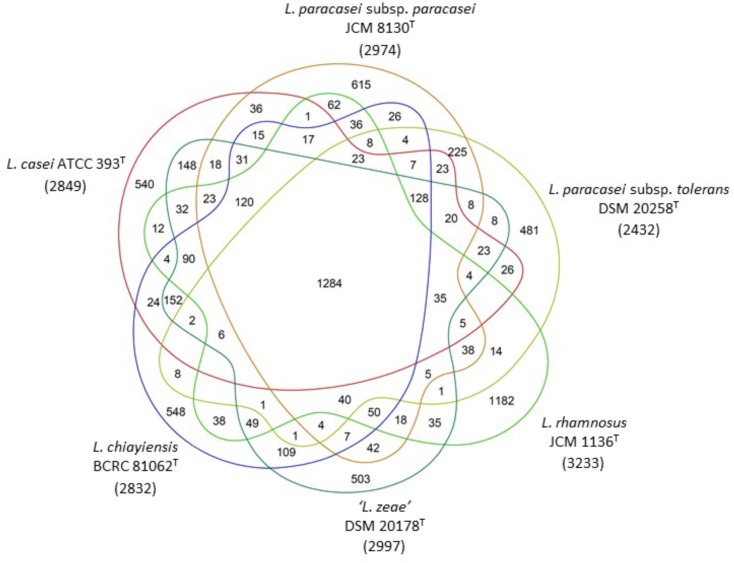
Ortholog analysis among six type strains in the *L. casei* group. Gene prediction and annotation were performed by RAST automated web server. Venn diagram showing the number of core, shared and unique genes for each genome. Numerals in parentheses under strain name are the numbers of the predicted ORFs.

**FIGURE 5:**
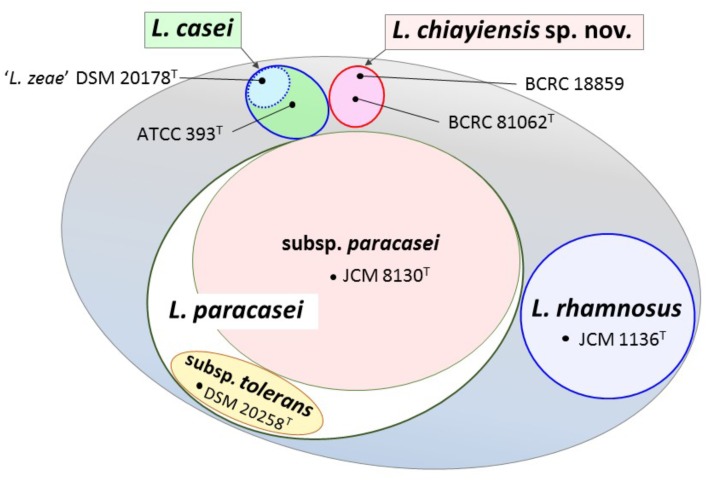
View showing the current state in classification of the *L. casei* group. The *L. casei* group has at least four distinct species; *L. casei, L. chiayiensis* sp. nov., *L. paracasei*, and *L. rhamnosus*. *L. paracasei* is comprised of two subspecies; *L. paracasei* subsp. *paracasei* and *L. paracasei* subsp. *tolerans*. ‘*L. zeae*’ has a possibility to be reclassified as the independent new taxon.

Venn diagram analysis based on genome sequences of six type strains in the *L. casei* group showed that 148 (4.9%) and 109 (3.6%) of the ORFs in ‘*L. zeae*’ DSM 20178^T^ were orthologous to those of *L. casei* ATCC 393^T^ and *L. chiayiensis* sp. nov. BCRC 81062^T^, respectively. This result indicated that ‘*L. zeae*’ DSM 20178^T^ is more closely related to these two strains than other strains (**Figure 4**). However, the results of polyphasic characterization implied that the current *L. casei* group has at least four distinct species; *L. casei, L. chiayiensis* sp. nov., *L. paracasei*, and *L. rhamnosus*. *L. paracasei* comprises two subspecies: *L. paracasei* subsp. *paracasei* and *L. paracasei* subsp. *tolerans* (**Figure 5**). The species ‘*L. zeae*’ was described by Dicks et al. (1996) and was thereafter rejected by the Judicial Commission of ICSP (Tindall, 2008). However, the ANI and dDDH values between *L. casei* ATCC 393^T^ and ‘*L. zeae*’ DSM 20178^T^, were 94.6 and 57.3%, respectively, which were lower than the species delineation threshold. This has a possibility that ‘*L. zeae*’ DSM 20178^T^ could be reclassified as the independent species or subspecies.

## Conclusion and Future Perspectives

Identifying closely related taxonomic groups (e.g., the *L. casei* group species) involves two steps. First, 16S rRNA gene sequencing is used to ascertain the species-group level of an unknown strain. This is subsequently the foundation used to define the housekeeping genes (*dnaJ, dnaK, mutL, pheS*, and *yycH*) used to ascertain the species level of the strain by MLSA. Group- and species-specific primers in combination with conventional PCR and SNaPshot minisequencing analyses are used; these techniques are useful for direct identification and differentiation of strains in the *L. casei* group, in particular, the multiplex primer set based on housekeeping gene is the simplest and quickest method. Nevertheless, proteomic identification of species within the *L. casei* group, based on the in-house database and applying ClinProTools to MALDI-TOF MS data, exhibits superior discriminatory power (at the subspecies level) and requires shorter analysis duration than DNA sequencing and PCR-based methods. This method also facilitates efficient quality control of probiotic products. Due to technological advancement, DNA sequencing can be performed inexpensively, and high-quality, useful bioinformatic tools are available for classifying and identifying prokaryotes. Whole-genome-based comparisons, such as ANI and dDDH, have become the gold standards for demarcation of phylogenetic relationships as well as for delineating new species; these methods can replace traditional DDH, conventional determination of DNA G+C content, and 16S rRNA and housekeeping genes sequencing.

## Author Contributions

C-HH analyzed the data and wrote the manuscript. S-WL analyzed the genome data. LH reviewed and revised the manuscript. KW designed this review, wrote the manuscript, and is a corresponding author. All authors read and approved the manuscript.

## Conflict of Interest Statement

The authors declare that the research was conducted in the absence of any commercial or financial relationships that could be construed as a potential conflict of interest.

## Footnotes

1 http://www.bacterio.net/lactobacillus.html
